# Association of low-level heavy metal exposure with risk of chronic kidney disease and long-term mortality

**DOI:** 10.1371/journal.pone.0315688

**Published:** 2024-12-17

**Authors:** Pai-Feng Kuo, Yun-Ting Huang, Min-Hsiang Chuang, Ming-Yan Jiang

**Affiliations:** 1 Department of Internal Medicine, Chi Mei Medical Center, Tainan, Taiwan; 2 Division of Nephrology, Department of Internal Medicine, Chi Mei Medical Center, Tainan, Taiwan; 3 Department of Pharmacy, Chia Nan University of Pharmacy & Science, Tainan, Taiwan; Guangdong Nephrotic Drug Engineering Technology Research Center, Institute of Consun Co. for Chinese Medicine in Kidney Diseases, CHINA

## Abstract

**Background:**

While the nephrotoxicity of lead and cadmium is well-established, the impact of low-level exposure on risk of chronic kidney disease (CKD) and long-term health outcomes, especially in CKD patients, remains unclear. This study examines the association between low-level lead and cadmium exposure with risks of CKD and long-term mortality.

**Method:**

We analyzed data from adult participants of 2003–2012 National Health and Nutrition Examination Survey in the United States. CKD was defined as estimated glomerular filtration rate < 60 ml/min/1.73 m^2^. Elevated blood lead (≥ 1.5 μg/dL) and cadmium (≥ 0.4 μg/L) levels were assessed for their associations with CKD and all-cause mortality, with survival tracked until December 31, 2019.

**Results:**

Among the 24,810 participants (mean age 44.4 years, 48.9% male), 1,309 (3.9%) had CKD. Lead and cadmium levels were significantly higher in participants with CKD compared to those without. Elevated lead (OR: 1.41, 95% CI: 1.15–1.74) and cadmium (OR: 1.23, 95% CI: 1.03–1.46) levels were both associated with increased CKD risk, with the highest risk in those with both lead ≥ 1.5 μg/dL and cadmium ≥ 0.4 μg/L (OR: 1.65, 95% CI 1.27–2.14). During a median follow-up of 141 months, 2,255 participants died (7.0 per 10,000 person-months). Elevated cadmium was associated with higher mortality risk in CKD (HR: 1.42, 95% CI: 1.07–1.88) and non-CKD populations (HR: 1.40, 95% CI: 1.24–1.58), while lead levels were not significantly associated with mortality in either group. Participants with both elevated lead and cadmium had a significantly higher mortality risk (HR: 1.32, 95% CI: 1.13–1.54).

**Conclusion:**

Low-level cadmium and lead exposure are linked to increased CKD risk, with cadmium also associated with higher long-term mortality in both CKD and non-CKD populations. These findings highlight the need for public health efforts to reduce exposure and further research on long-term impacts.

## Introduction

Heavy metal exposure, particularly to lead and cadmium, poses significant public health risks due to their bioaccumulative and toxic effects across multiple organ systems [1, 2]. Lead, a widespread environmental contaminant, has been linked to a range of health issues, including damage to the central nervous system, pulmonary dysfunction, anemia, gastrointestinal disturbances, hepatic injury, and cardiovascular diseases [1, 2]. A key concern is its nephrotoxicity, which can manifest as both acute and chronic kidney damage [3]. Acute lead nephrotoxicity typically results from direct injury to the proximal tubules, while chronic exposure, even at low levels, can lead to chronic kidney disease (CKD), characterized by decreased estimated glomerular filtration rate (eGFR), mild proteinuria, and often normal urine sediment. Chronic exposure can also result in interstitial nephritis, leading to progressive renal dysfunction [3].

Cadmium exposure, another environmental and occupational hazard, similarly leads to adverse health outcomes, including bone disease, renal dysfunction, hepatic damage, gastrointestinal disorders, pulmonary injuries, and malignancies [2]. Cadmium-induced nephrotoxicity is typically characterized by proximal tubular dysfunction, evident through clinical markers such as glucosuria, aminoaciduria, and low molecular weight proteinuria [3]. Its accumulation in the renal cortex is of particular concern, as it contributes to chronic tubular and glomerular damage, resulting in progressive renal dysfunction [3].

Although the nephrotoxic effects of lead and cadmium are well-documented, the association between low-level exposure and the risk of CKD remains debated. Epidemiological studies have yielded inconsistent findings. For instance, a health survey in Southern Taiwan reported a significant association between elevated blood lead levels and increased risks of proteinuria and reduced eGFR (below 60 mL/min/1.73 m^2^) [4]. In the United States (U.S.), elevated blood lead and cadmium levels were correlated with decreased eGFR; however, after adjustments, only lead levels showed a significant association with CKD [5]. In contrast, a study in South Korea found no significant association between blood metal levels and CKD in the general population, although cadmium was linked to CKD among individuals with comorbid hypertension or diabetes [6]. Furthermore, among CKD patients, there was no observed link between blood levels of lead and cadmium and the progression to end-stage renal disease (ESRD) or mortality [7].

These inconsistent findings highlight the complexity of the relationship between low-level heavy metal exposure and renal health. While cross-sectional studies have suggested a potential link between low-level metal exposure and kidney disease, there is a paucity of longitudinal studies examining these associations, particularly concerning long-term health outcomes in populations with compromised renal function. To address this gap, our study uses a nationally representative U.S. population to examine the association between low-level lead and cadmium exposure and the risk of CKD, as well as their relationship with long-term mortality. This research aims to provide clearer insights into the public health implications of heavy metal exposure, particularly for populations vulnerable to kidney disease.

## Methods

### Data source

This study utilized data from the National Health and Nutrition Examination Survey (NHANES) in the U.S., conducted by the National Center for Health Statistics (NCHS). NHANES is a series of cross-sectional surveys designed to assess the health and nutritional status of the U.S. general population through multistage probability sampling of the non-institutionalized civilian population. The survey includes health-related questionnaires, physical examinations, and laboratory tests. Data are released in 2-year cycles and are publicly accessible through the NCHS website (https://wwwn.cdc.gov/nchs/nhanes/Default.aspx). All NHANES protocols received ethical approval from the NCHS Research Ethics Review Board, and written informed consent was obtained from all participants. This study adhered to the ethical principles of the Declaration of Helsinki.

### Study population

We combined five consecutive NHANES cycles (2003–2004 to 2011–2012) to form the study population, initially comprising 50,912 individuals. This time frame was selected to ensure consistency in laboratory methodology and sample eligibility criteria. Starting in 2003, NHANES adopted inductively coupled plasma mass spectrometry (ICP-MS), a more sensitive and precise method for measuring blood lead and cadmium than previous techniques. In 2013–2014 NHANES, sample eligibility for lead and cadmium testing changed to include only a half-sample of participants aged 12 and older. To maintain consistency, we therefore restricted our study to 2003–2012 data. Participants were excluded if they were under 18 years of age (n = 21,110), over 80 years (n = 2,138), had undergone dialysis in the previous 12 months (n = 87), or lacked data on serum creatinine (n = 2,746) or blood lead and cadmium levels (n = 21). After exclusions, the final study cohort consisted of 24,810 individuals.

### Exposure variables

Whole blood levels of lead and cadmium, the exposure variables in our study, were measured using inductively coupled plasma mass spectrometry (ICP-MS), a multi-element analytical technique utilizing quadrupole ICP-MS technology. Blood lead levels were categorized into two groups: <1.5 μg/dL and ≥1.5 μg/dL, while blood cadmium levels were classified as <0.4 μg/L and ≥0.4 μg/L. These cutoff levels were selected to create balanced groups and facilitate analysis, even though they are below the established toxic thresholds of 3.5 μg/dL for lead [8] and 5 μg/L for cadmium [9]. However, epidemiological studies have reported adverse health outcomes at progressively lower levels of exposure [8, 9]. These findings suggest potential subclinical effects at lower concentrations, thus justifying the use of these lower cut-off values to capture potential risks that may not be immediately apparent through conventional thresholds.

### Outcome variables

The outcome variable, chronic kidney disease (CKD), is defined as having an estimated glomerular filtration rate (eGFR) below 60 mL/min/1.73 m^2^. Since lead and cadmium are primarily excreted in urine, reduced eGFR may impair the elimination of these metals, potentially resulting in elevated blood levels that reflect kidney dysfunction rather than causation of CKD. To mitigate the possibility of reverse causality, we conducted additional analyses using albuminuria (defined as a urinary albumin-creatinine ratio [ACR] ≥30 mg/g) as an outcome in individuals with eGFR ≥ 60 mL/min/1.73 m^2^. Serum and urine creatinine concentrations were measured using the Jaffe rate method (kinetic alkaline picrate) on the DxC800 modular chemistry analyzer, which is standardized against an isotope dilution mass spectrometry (IDMS) reference method. Urinary albumin levels were determined using a solid-phase fluorescent immunoassay, a non-competitive, double-antibody method designed to measure human albumin in urine. The eGFR was calculated using the 2021 Chronic Kidney Disease Epidemiology Collaboration (CKD-EPI) equation.

Mortality status was determined by linking NHANES data to the National Death Index (NDI) through probabilistic matching and death certificate review. Follow-up continued from the baseline NHANES interview until death or December 31, 2019, whichever occurred first.

### Covariates

Demographic and clinical covariates included self-reported race/ethnicity (categorized as non-Hispanic White, non-Hispanic Black, Hispanic, or other, including multiracial individuals), smoking status (never, former, current), education level (high school or below vs. some college or above), and marital status [non-single (married or living with a partner) vs. single (widowed, divorced, separated, or never married)]. The family income-to-poverty ratio was calculated by dividing family income by the poverty threshold for that family’s size, year, and state. Body mass index (BMI) was calculated by dividing body weight in kilograms by height in meters squared. Diabetes mellitus and hypertension were defined by self-reported diagnosis or medication use. Cardiovascular disease was defined by a self-reported history of congestive heart failure, coronary heart disease, angina, or heart attack. Previous stroke was also self-reported.

### Statistical analysis

NHANES sample weights were applied to account for non-response, oversampling, and non-coverage, ensuring the data remained representative of the U.S. population. Continuous variables were presented as survey-weighted means ± standard error (SE) and were compared using independent t-tests. Categorical variables were expressed as counts and survey-weighted proportions, with differences assessed using χ^2^ tests. A p-value of <0.05 was considered statistically significant for all analyses.

Weighted logistic regression was performed to assess the association between elevated blood lead and cadmium levels and CKD, adjusting for age, sex, race/ethnicity, BMI, diabetes, hypertension, cardiovascular disease, history of stroke, smoking status, education level, marital status, and family income-to-poverty ratio. Results were presented as odds ratios (OR) with 95% confidence intervals (CI). To assess potential interaction effects between lead and cadmium on CKD risk, participants were categorized into four groups based on their lead and cadmium levels: group 1 (Pb < 1.5 μg/dL and Cd < 0.4 μg/L), group 2 (Pb ≥ 1.5 μg/dL and Cd < 0.4 μg/L), group 3 (Pb < 1.5 μg/dL and Cd ≥ 0.4 μg/L), and group 4 (Pb ≥ 1.5 μg/dL and Cd ≥ 0.4 μg/L). Additionally, to address reverse causality, a sensitivity analysis was conducted using albuminuria (urinary ACR ≥ 30 mg/g) as the outcome among participants with eGFR ≥ 60 mL/min/1.73 m^2^.

Survival analyses were conducted using Kaplan-Meier curves and Log-Rank tests to examine the association between blood lead or cadmium levels and mortality among individuals with and without CKD. Additionally, weighted Cox proportional hazards regression was used to explore the relationship between lead and cadmium levels and mortality risk, adjusting for age, sex, race/ethnicity, BMI, eGFR, diabetes, hypertension, cardiovascular disease, history of stroke, smoking status, education level, marital status, and family income-to-poverty ratio. Hazard ratios (HR) with 95% CI were reported. Statistical interaction between lead and cadmium on mortality risk was assessed, with adjustment made for age, sex, race/ethnicity, BMI, diabetes, hypertension, CKD, albuminuria, smoking status, education level, marital status, and family income-to-poverty ratio. All analyses were performed using SAS version 9.4 (SAS Institute, Cary, NC, USA).

## Results

The study population had a weighted mean age of 44.4 ± 0.3 years, with 48.9% being male. The racial/ethnic distribution was 69.3% White, 11.1% Black, 13.4% Hispanic, and 6.2% from other racial/ethnic groups (Table 1). The overall weighted prevalence of elevated lead levels (≥ 1.5 μg/dL) was 39.0% (Table 1). Participants with lead levels ≥ 1.5 μg/dL were more likely to be male, older, have a lower BMI, lower educational attainment, lower family income-to-poverty ratio, be married or living with a partner, be former or current smokers, and have higher rates of comorbidities such as hypertension, cardiovascular disease, and stroke compared to those with lead levels < 1.5 μg/dL.

**Table 1:** Baseline characteristics of the study population.

		Lead			Cadmium		
	Total	< 1.5μg/dL	≥ 1.5μg/dL	p	< 0.4μg/L	≥ 0.4μg/L	p
**No. of participants**	24810	14495 (61.0)	10315 (39.0)		14380 (59.7)	10430 (40.3)	
**Male**	12150 (48.9)	5686 (40.0)	6464 (62.1)	< 0.001	7366 (52.4)	4784 (43.7)	< 0.001
**Age**	44.4±0.3	40.0±0.3	51.4±0.3	< 0.001	42.4±0.3	47.5±0.3	< 0.001
**Race**				< 0.01			< 0.001
White	10951 (69.3)	6468 (70.0)	4483 (68.2)		6135 (69.7)	4816 (68.6)	
Black	5465 (11.1)	3076 (10.8)	2389 (11.7)		3050 (10.3)	2415 (12.4)	
Hispanics	6673 (13.4)	3963 (13.7)	2710 (12.9)		4453 (15.6)	2220 (10.2)	
Others	1721 (6.2)	988 (5.6)	733 (7.2)		742 (4.5)	979 (8.7)	
**BMI**	28.5±0.1	28.8±0.1	28.1±0.1	< 0.001	29.0±0.1	27.9±0.1	< 0.001
**Education level**				< 0.001			< 0.001
≤ high school	11581 (41.2)	5706 (35.9)	5875 (49.2)		5800 (34.7)	5781 (50.7)	
≥ some college	11387 (58.8)	7215 (64.1)	4172 (50.8)		7210 (65.3)	4177 (49.3)	
**Marital status**				< 0.01			< 0.001
Non-single	14278 (64.5)	7915 (63.3)	6363 (66.5)		8446 (66.7)	5832 (61.4)	
Single	9722 (35.5)	5838 (36.7)	3884 (33.5)		5311 (33.3)	4411 (38.6)	
**Income-poverty ratio**	3.00±0.04	3.05±0.04	2.93±0.04	< 0.001	3.19±0.04	2.73±0.04	< 0.001
**Smoking status**				< 0.001			< 0.001
Never	12244 (52.8)	8089 (61.5)	4155 (39.9)		9310 (70.9)	2934 (26.7)	
Former	5438 (24.1)	2604 (21.0)	2834 (28.7)		3105 (24.8)	2333 (22.9)	
Current	5287 (23.1)	2231 (17.5)	3056 (31.4)		597 (4.3)	4690 (50.4)	
**Comorbidity**							
Diabetes	2639 (7.9)	1445 (7.8)	1194 (8.1)	0.47	1486 (7.9)	1153 (8.0)	0.79
Hypertension	7530 (28.0)	3589 (23.8)	3941 (34.6)	< 0.001	3898 (25.5)	3632 (31.7)	< 0.001
CVD	1648 (5.8)	614 (3.8)	1034 (8.8)	< 0.001	691 (4.2)	957 (8.1)	< 0.001
Stroke	715 (2.3)	259 (1.5)	456 (3.4)	< 0.001	280 (1.5)	435 (3.4)	< 0.001

Note: Continuous variables were presented as mean ± standard error. Categorical variables were presented as count (weighted proportion).

Abbreviations: BMI: body mass index; CVD: cardiovascular disease.

Similarly, the weighted prevalence of elevated cadmium levels (≥ 0.4 μg/L) was 40.3% (Table 1). Participants with cadmium levels ≥ 0.4 μg/L were more likely to be female, older, have a lower BMI, lower educational attainment, lower family income-to-poverty ratio, be single (widowed, divorced, separated, or never married), be current smokers, and have higher rates of hypertension, cardiovascular disease, and stroke compared to those with cadmium levels < 0.4 μg/L.

Among the 24,810 participants, 1,309 had CKD, corresponding to a weighted prevalence of 3.9% (Table 2). The weighted mean lead level was significantly higher in participants with CKD compared to those without (2.14 ± 0.05 μg/dL vs. 1.58 ± 0.02 μg/dL, p < 0.001). Similarly, cadmium levels were higher in participants with CKD compared to those without CKD (0.60 ± 0.02 μg/L vs. 0.53 ± 0.01 μg/L, p < 0.01). Participants with lead levels ≥ 1.5 μg/dL and those with cadmium levels ≥ 0.4 μg/L were more likely to have CKD than those with lower levels of these metals. After adjusting for potential confounders (age, sex, race, BMI, diabetes, hypertension, cardiovascular disease, stroke, smoking status, education, marital status, and family income-to-poverty ratio), elevated blood lead (OR: 1.41, 95% CI 1.15–1.74, p < 0.01) and cadmium (OR: 1.23, 95% CI 1.03–1.46, p < 0.05) levels were both independently associated with increased CKD risk (Table 2). The highest risk was observed in participants with both lead ≥ 1.5 μg/dL and cadmium ≥ 0.4 μg/L (group 4) (OR: 1.65, 95% CI 1.27–2.14, p < 0.001). However, we did not observe a significant multiplicative interaction between lead and cadmium (p for interaction > 0.05) (Table 2).

**Table 2:** Association of lead (Pb) and cadmium (Cd) levels with risk of chronic kidney disease (CKD).

	CKD (n = 1309, 3.9%)	Non-CKD (n = 23501, 96.1%)	Crude OR (95% CI)	Age- and sex- adjusted OR (95% CI)	Fully adjusted OR (95% CI) ^#^
Pb (μg/dL)	2.14±0.05	1.58±0.02	1.12 (1.08–1.16) ***	1.05 (1.01–1.08) *	1.06 (1.03–1.10) ***
Pb < 1.5	432 (36.6%)	14063 (62.0%)	1	1	1
Pb ≥ 1.5	877 (63.4%)	9438 (38.0%)	2.83 (2.40–3.34) ***	1.28 (1.07–1.53) **	1.41 (1.15–1.74) **
Cd (μg/L)	0.60±0.02	0.53±0.01	1.16 (1.07–1.25) ***	1.13 (1.01–1.27) *	1.26 (1.12–1.41) ***
Cd < 0.4	562 (45.2%)	13818 (60.3%)	1	1	1
Cd ≥ 0.4	747 (54.8%)	9683 (39.7%)	1.84 (1.58–2.13) ***	1.13 (0.96–1.34)	1.23 (1.03–1.46) *
Pb × Cd ^§^					
Group 1	228 (20.3%)	9649 (43.3%)	1	1	1
Group 2	334 (24.9%)	4169 (17.0%)	3.13 (2.51–3.91) ***	1.35 (1.07–1.72) *	1.52 (1.14–2.01) **
Group 3	204 (16.2%)	4414 (18.7%)	1.85 (1.45–2.37) ***	1.19 (0.92–1.54)	1.32 (0.99–1.76)
Group 4	543 (38.5%)	5269 (21.1%)	3.90 (3.18–4.78) ***	1.39 (1.10–1.76) **	1.65 (1.27–2.14) ***
*P*_interaction_			> 0.05	> 0.05	> 0.05

^#^: adjusted for age, sex race, body mass index, diabetes, hypertension, cardiovascular disease, previous stroke, smoking status, educational level, marital status, and family income to poverty ratio.

^§^: Group 1: Pb < 1.5 μg/dL and Cd < 0.4 μg/L (n = 9877); Group 2: Pb ≥ 1.5 μg/dL and Cd < 0.4 μg/L (n = 4503); Group 3: Pb < 1.5 μg/dL and Cd ≥ 0.4 μg/L (n = 4618); Group 4: Pb ≥ 1.5 μg/dL and Cd ≥ 0.4 μg/L (n = 5812).

*: p<0.05;

**: p<0.01;

***: p<0.001.

Among participants with eGFR ≥ 60 mL/min/1.73 m^2^, 2,159 had albuminuria. We found that higher blood lead levels were associated with an increased risk of albuminuria, with an adjusted OR of 1.04 (95% CI: 1.01–1.07) per 1 μg/dL increment (p < 0.05) (S1 Table). Similarly, higher blood cadmium levels were linked to an elevated risk of albuminuria, with an adjusted OR of 1.15 (95% CI: 1.03–1.27) per 1 μg/L increment (p < 0.05). Cadmium levels ≥ 0.4 μg/L were significantly associated with an increased risk of albuminuria (adjusted OR: 1.35, 95% CI: 1.16–1.57, p < 0.001), whereas lead levels ≥ 1.5 μg/dL were not significantly associated with albuminuria after adjustment for potential confounders (S1 Table).

**Fig 1:**
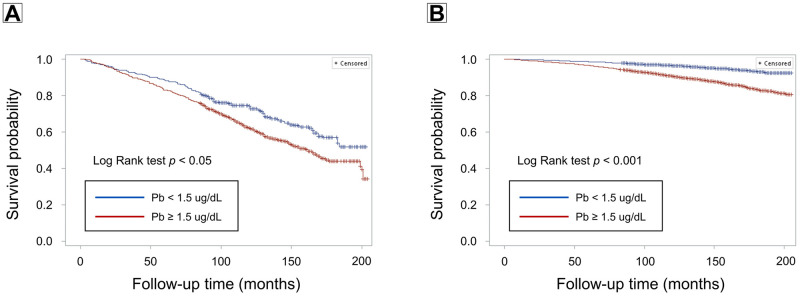
Survival curves showing the association of lead (Pb) levels (< 1.5 vs. ≥ 1.5 μg/dL) with mortality risk among individuals with (A) and without (B) chronic kidney disease (CKD).

**Fig 2:**
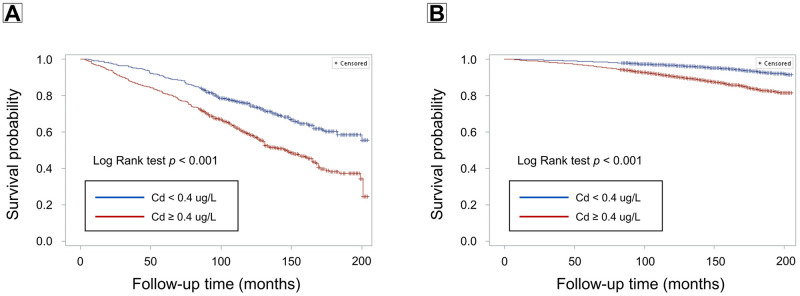
Survival curves showing the association of cadmium (Cd) levels (< 0.4 vs. ≥ 0.4 μg/L) with mortality risk among individuals with (A) and without (B) chronic kidney disease (CKD).

During a median follow-up of 141 months (interquartile range: 115–172 months), 2,255 participants died, corresponding to a crude death rate of 7.0 per 10,000 person-months. Among the 1,309 participants with CKD, 602 died, a crude death rate of 41.6 per 1,0000 person-months. In participants with CKD, elevated blood lead (Log Rank test p < 0.05) and cadmium levels (Log Rank test p < 0.001) were both associated with increased mortality (Figs 1A and 2A). Similarly, in participants without CKD, elevated lead (Log Rank p < 0.001) and cadmium levels (Log Rank p < 0.001) were linked to higher mortality risk (Figs 1B and 2B). After multivariable adjustment, elevated blood cadmium was associated with a 42% increased risk of death in the CKD population (HR: 1.42, 95% CI: 1.07–1.88, p < 0.05) and a 40% increased risk in the non-CKD population (HR: 1.40, 95% CI: 1.24–1.58, p < 0.001) (Table 3). However, no significant association between elevated blood lead levels and mortality risk was found in either group (Table 3). Notably, participants with both elevated lead (≥ 1.5 μg/dL) and cadmium (≥ 0.4 μg/L) had a significantly increased mortality risk (HR: 1.32, 95% CI: 1.13–1.54, p < 0.001) (S2 Table), with evidence of a significant synergistic effect between lead and cadmium (p for interaction < 0.01).

**Table 3:** Association of lead (Pb) and cadmium (Cd) levels with mortality risk among individuals with and without chronic kidney disease (CKD).

	Model 1	Model 2
	HR (95% CI)	HR (95% CI)
**CKD population**		
Pb (every 1-unit increment)	1.07 (1.03–1.11) **	1.02 (0.96–1.08)
Pb < 1.5 ug/dL	1	1
Pb ≥ 1.5 ug/dL	1.23 (0.93–1.61)	1.18 (0.84–1.65)
Cd (every 1-unit increment)	1.52 (1.24–1.86) ***	1.41 (1.11–1.79) **
Cd < 0.4 ug/L	1	1
Cd ≥ 0.4 ug/L	1.67 (1.34–2.08) ***	1.42 (1.07–1.88) *
**Non-CKD population**		
Pb (every 1-unit increment)	1.05 (1.03–1.07) ***	1.02 (1.00–1.05)
Pb < 1.5 ug/dL	1	1
Pb ≥ 1.5 ug/dL	1.16 (1.04–1.29) *	0.98 (0.86–1.11)
Cd (every 1-unit increment)	1.48 (1.41–1.55) ***	1.23 (1.15–1.32) ***
Cd < 0.4 ug/L	1	1
Cd ≥ 0.4 ug/L	2.00 (1.79–2.25) ***	1.40 (1.24–1.58) ***

Model 1: adjusted for age and sex.

Model 2: adjusted for age, sex race, body mass index, estimated glomerular filtration rate (eGFR), diabetes, hypertension, cardiovascular disease, previous stroke, smoking status, educational level, marital status, and family income to poverty ratio.

*: p<0.05;

**: p<0.01;

***: p<0.001.

## Discussion

In this study, we examined the associations between low-level exposure to lead and cadmium and the risk of CKD and all-cause mortality in a large, nationally representative U.S. population. Our findings indicate that elevated blood lead (≥ 1.5 μg/dL) and cadmium (≥ 0.4 μg/L) levels were both significantly associated with an increased risk of CKD. Additionally, elevated cadmium levels were linked to a higher risk of all-cause mortality in individuals with and without CKD, while elevated lead levels were not significantly associated with mortality risk. Notably, participants with concurrent elevations in both lead and cadmium (Pb ≥ 1.5 μg/dL and Cd ≥ 0.4 μg/L) exhibited a significantly higher mortality risk, demonstrating a synergistic interaction between the two metals. These findings support and expand the growing body of evidence linking even low-level heavy metal exposure to adverse health outcomes [10], highlighting potential risks at levels traditionally considered below toxic thresholds.

In our study, the association between elevated heavy metal levels and CKD remained significant even after adjusting for potential confounders such as age, sex, race, BMI, and known cardiovascular risk factors, underscoring the independent role of lead exposure in kidney disease development [10]. While blood lead levels in the U.S. population have decreased since the phase-out of leaded gasoline and lead pipes [11], our findings highlight that even low-level exposures continue to pose risks to kidney health. These results are consistent with previous research linking lead exposure to nephrotoxicity through mechanisms such as oxidative stress, inflammation, and tubulointerstitial damage [12, 13]. Given the persistence of lead in the environment and its potential for bioaccumulation, public health efforts to further reduce lead exposure remain critical, particularly for vulnerable populations such as those with pre-existing kidney disease or socioeconomic disadvantages.

One of the key findings of our study was the association between elevated cadmium levels and increased mortality risk in both CKD and non-CKD populations. This suggests that cadmium not only accelerates kidney dysfunction but also contributes to broader systemic effects that raise mortality risk, potentially through its role in cardiovascular disease, cancer, and impaired immune function [12, 14, 15]. While cadmium exposure is primarily linked to smoking and industrial emissions, dietary sources such as contaminated vegetables and grains also contribute to its accumulation [16]. This highlights the need for continued surveillance of cadmium exposure sources and interventions to mitigate its impact, particularly among smokers, who were overrepresented in the elevated cadmium group in our study. Previous research has identified cadmium as a potent environmental toxin linked to cardiovascular disease, cancer, and premature death [15]. Our results support and extend this evidence by demonstrating that even low-level cadmium exposure significantly increases mortality risk, independent of traditional risk factors.

Interestingly, while elevated lead levels were associated with CKD, no significant association with mortality was observed in either the CKD or non-CKD groups. This finding contrasts with some earlier studies that reported a link between lead exposure and increased mortality risk [17], particularly from cardiovascular causes [12, 17]. The lack of a significant mortality association in our study may be due to lead’s effects on mortality being mediated through the development of CKD, which we have already accounted for in our analysis, or through other pathways, such as cardiovascular events, which may be underpowered in our population. Additional factors, such as variations in population characteristics, exposure duration, or the intensity of lead exposure, may also contribute to these findings and warrant further investigation.

The findings that both lead and cadmium exposure were more common among individuals with lower socioeconomic status, lower educational attainment, and higher rates of smoking and comorbidities such as hypertension and cardiovascular disease highlight the disproportionate burden of heavy metal exposure among vulnerable populations [18, 19]. These social determinants likely exacerbate the health impacts of lead and cadmium, compounding their effects on kidney function and mortality. Public health interventions targeting at-risk populations may help mitigate these exposures and their downstream health consequences.

The strengths of our study include the use of a large, nationally representative cohort, the robust assessment of lead and cadmium exposure using reliable biomarkers, and the long follow-up period for mortality outcomes. However, several limitations should be noted. First, the cross-sectional design limits our ability to infer causality between low-level heavy metal exposure and CKD risk; longitudinal studies are needed to better establish temporal relationships. Nevertheless, the observed significant associations between albuminuria and low-level lead or cadmium exposure in participants with preserved kidney function help address concerns about reverse causality, suggesting that these findings are not solely a result of impaired kidney function impacting metal levels. Second, lead and cadmium levels were measured at a single time point, which may not adequately reflect cumulative lifetime exposure. Third, as this is an observational study, there is a risk of confounding and survivor bias, limiting our ability to definitively establish a causal relationship between heavy metal exposure and CKD or mortality. Although we adjusted for many confounders, the possibility of residual confounding remains. For example, we were unable to account for certain dietary or behavioral factors that may influence the association between metal levels and CKD or mortality. Fourth, the exposure cutoffs for lead and cadmium are somewhat arbitrary, and the potential health impacts of even lower exposure levels warrant further investigation.

Our findings have important public health implications. While epidemiological evidence continues to reveal health risks at progressively lower blood lead and cadmium levels, our results underscore the need to address even low-level exposures. Given the associations of lead and cadmium with CKD and mortality, and particularly the synergistic effect observed when both metals are present at elevated levels, efforts to further reduce environmental exposure to these metals should remain a priority. This is especially pertinent for individuals at higher risk, such as smokers and those with lower socioeconomic status, who may be disproportionately exposed. Enhancing regulations on industrial emissions, improving dietary safety, and promoting smoking cessation are crucial strategies to mitigate the long-term health effects of lead and cadmium [20].

In conclusion, our study provides evidence that even low-level lead and cadmium exposures are associated with an increased risk of CKD, and elevated cadmium levels are further associated with higher all-cause mortality. Notably, we observed a synergistic effect between lead and cadmium exposure on mortality risk, highlighting the compounded health burden when both metals are present at elevated levels. These findings underscore the need for continued efforts to reduce exposure to heavy metals, particularly in vulnerable populations, to lessen the burden of CKD and improve long-term health outcomes. Future research should focus on longitudinal studies to clarify the causal pathways and further explore the combined impacts of low-level lead and cadmium exposure on CKD and mortality.

## Supporting information

S1 Table Association of lead (Pb) and cadmium (Cd) levels with risks of albuminuria in participants with estimated glomerular filtration rate ≥ 60 ml/min/1.73 m^2^. (DOCX)

S2 Table Risk factors for mortality in the total study population. (DOCX)
