# Supplementary material for: Association of low-level heavy metal exposure with risk of chronic kidney disease and long-term mortality
Source: PLoS One. 2024 Dec 17;19(12):e0315688. doi: 10.1371/journal.pone.0315688 (PMC11651581; doi:10.1371/journal.pone.0315688)
Supplement: S1 Table — (DOCX) [file pone.0315688.s001.docx]

**Supplemental Table S1.** Association of lead (Pb) and cadmium (Cd) levels with risks of albuminuria in participants with estimated glomerular filtration rate ≥ 60 ml/min/1.73 m²

|  | Crude OR  (95% CI) | Age- and sex- adjusted OR (95% CI) | Fully adjusted OR (95% CI) ^#^ |
| --- | --- | --- | --- |
| Pb (µg/dL) | 1.06 (1.03-1.09) ^***^ | 1.04 (1.01-1.07) ^*^ | 1.04 (1.01-1.07) ^*^ |
| Pb < 1.5 | 1 | 1 | 1 |
| Pb ≥ 1.5 | 1.17 (1.03-1.33) ^*^ | 0.97 (0.84-1.12) | 0.99 (0.86-1.14) |
|  |  |  |  |
| Cd (µg/L) | 1.23 (1.15-1.32) ^***^ | 1.21 (1.12-1.31) ^***^ | 1.15 (1.03-1.27) ^*^ |
| Cd < 0.4 | 1 | 1 | 1 |
| Cd ≥ 0.4 | 1.52 (1.37-1.67) ^***^ | 1.38 (1.24-1.52) ^***^ | 1.35 (1.16-1.57) ^***^ |

^#^: adjusted for age, sex race, body mass index, diabetes, hypertension, cardiovascular disease, previous stroke, smoking status, educational level, marital status, and family income to poverty ratio.

^*^: p<0.05; ^**^: p<0.01; ^***^: p<0.001.
