# Supplementary material for: Association of low-level heavy metal exposure with risk of chronic kidney disease and long-term mortality
Source: PLoS One. 2024 Dec 17;19(12):e0315688. doi: 10.1371/journal.pone.0315688 (PMC11651581; doi:10.1371/journal.pone.0315688)
Supplement: S2 Table — (DOCX) [file pone.0315688.s002.docx]

**Supplemental Table S2.** Risk factors for mortality in the total study population

|  | Hazard ratio (95% CI) | *p* value |
| --- | --- | --- |
| **Pb × Cd** ^§^ |  |  |
| Group 1 | 1 |  |
| Group 2 | 0.84 (0.70-1.01) | 0.06 |
| Group 3 | 1.17 (0.98-1.40) | 0.08 |
| Group 4 | 1.32 (1.13-1.54) | < 0.001 |
| **Age** | 1.08 (1.08-1.09) | < 0.001 |
| **Male (vs. female)** | 1.78 (1.61-1.96) | < 0.001 |
| **Race** |  |  |
| White |  |  |
| Black | 0.87 (0.77-0.99) | < 0.05 |
| Hispanics | 0.62 (0.53-0.73) | < 0.001 |
| Others | 0.65 (0.49-0.87) | < 0.01 |
| **BMI** | 1.01 (1.00-1.01) | 0.12 |
| **Diabetes** | 1.47 (1.33-1.63) | < 0.001 |
| **Hypertension** | 1.20 (1.07-1.35) | < 0.01 |
| **CKD** | 1.37 (1.18-1.60) | < 0.001 |
| **Albuminuria** | 1.96 (1.73-2.22) | < 0.001 |
| **Smoking status** |  |  |
| Never | 1 |  |
| Former | 1.18 (1.02-1.35) | < 0.05 |
| Current | 1.79 (1.56-2.04) | < 0.001 |
| **Education level** |  |  |
| ≤ high school |  |  |
| ≥ some college | 0.91 (0.80-1.03) | 0.12 |
| **Marital status** |  |  |
| Non-single |  |  |
| Single | 1.35 (1.21-1.51) | < 0.001 |
| **Income-poverty ratio** | 0.84 (0.81-0.88) | < 0.001 |

Abbreviations: Pb: lead; Cd: cadmium; BMI: body mass index; CKD: chronic kidney disease (estimated glomerular filtration rate < 60 mL/min/1.73 m^2^)

^§^: group 1: Pb < 1.5 µg/dL and Cd < 0.4 µg/L; group 2: Pb ≥ 1.5 µg/dL and Cd < 0.4 µg/L; group 3: Pb < 1.5 µg/dL and Cd ≥ 0.4 µg/L; group 4: Pb ≥ 1.5 µg/dL and Cd ≥ 0.4 µg/L.
